# Low Physical Activity and Its Association with Diabetes and Other Cardiovascular Risk Factors: A Nationwide, Population-Based Study

**DOI:** 10.1371/journal.pone.0160959

**Published:** 2016-08-17

**Authors:** Laura Brugnara, Serafín Murillo, Anna Novials, Gemma Rojo-Martínez, Federico Soriguer, Albert Goday, Alfonso Calle-Pascual, Luis Castaño, Sonia Gaztambide, Sergio Valdés, Josep Franch, Conxa Castell, Joan Vendrell, Roser Casamitjana, Anna Bosch-Comas, Elena Bordiú, Rafael Carmena, Miguel Catalá, Elias Delgado, Juan Girbés, Alfonso López-Alba, Maria Teresa Martínez-Larrad, Edelmiro Menéndez, Inmaculada Mora-Peces, Gemma Pascual-Manich, Manuel Serrano-Ríos, Ramon Gomis, Emilio Ortega

**Affiliations:** 1 CIBERDEM—Spanish Biomedical Research Centre in Diabetes and Associated Metabolic Disorders, Madrid, Spain; 2 IDIBAPS—August Pi i Sunyer Biomedical Research Institute / Hospital Clínic de Barcelona, Barcelona, Spain; 3 Hospital Universitario Carlos Haya, Department of Endocrinology and Nutrition, Málaga, Spain; 4 Hospital del Mar, Department of Endocrinology and Nutrition, Barcelona, Spain; 5 Hospital Universitario San Carlos, Madrid, Spain; 6 Hospital Universitario de Cruces, UPV-EHU, Diabetes Research Group, Baracaldo, Spain; 7 EAP Raval Sud, Institut Català de la Salut, Red GEDAPS, IDIAP, Barcelona, Spain; 8 Public Health Division, Autonomous Government of Catalonia, Barcelona, Spain; 9 Department of Endocrinology and Nutrition, Hospital Universitario Joan XXIII, Tarragona, Spain; 10 Department of Medicine and Endocrinology, Hospital Clínico Universitario de Valencia, Valencia, Spain; 11 Department of Endocrinology and Nutrition, Hospital Universitario Central de Asturias, Facultad de Medicina y Ciencias de la Salud, Universidad de Oviedo, Oviedo, Spain; 12 Hospital Arnau de Vilanova, Valencia, Spain; 13 Fundación Hospital de Jove, Gijón, Spain; 14 Instituto de Investigación Sanitaria del Hospital Clínico San Carlos (IdISSC), Madrid, Spain; 15 Canarian Health Service, San Cristóbal de la Laguna, Tenerife, Spain; 16 CIBEROBN—Spanish Biomedical Research Centre in Physiopathology of Obesity; 17 Department of Endocrinology and Nutrition, ICMDM, Hospital Clinic Barcelona; Universitat de Valencia, SPAIN

## Abstract

Low physical activity (PA), or sedentary lifestyle, is associated with the development of several chronic diseases. We aimed to investigate current prevalence of sedentariness and its association with diabetes and other cardiovascular risk factors. PA was evaluated in a population-based, cross-sectional, randomly sampled study conducted in 2009–2010 in Spain. International Physical Activity Questionnaire (SF-IPAQ) was used to assess PA. 4991 individuals (median age 50 years, 57% women) were studied. Prevalence of sedentariness was 32.3% for men and 39% for women (p < 0.0001). Sex differences were particularly notable (age*sex interaction, p = 0.0024) at early and older ages. Sedentary individuals had higher BMI (28 vs. 27 kg/m^2^) and obesity prevalence (37 vs. 26%). Low PA was present in 44, 43, and 38% of individuals with known diabetes (KDM), prediabetes/unknown-diabetes (PREDM/UKDM), and normal glucose regulation (p = 0.0014), respectively. No difference between KDM and PREDM/UKDM (p = 0.72) was found. Variables independently associated (p < 0.05) with sedentariness were age, sex, BMI, central obesity, Mediterranean diet adherence, smoking habit, HDL-cholesterol, triglycerides and dyslipidemia. Low PA is on the rise in Spain, especially among women. Sedentariness is associated with several cardiovascular risk factors and may be responsible for the increasing prevalence of obesity and diabetes in this country.

## Introduction

The 2010 World Health Report describes how a few major risk factors account for much of the morbidity and mortality across the globe. Sedentary lifestyle, or low physical activity (PA), is among the leading causes of the major noncommunicable diseases, including cardiovascular disease, type 2 diabetes and diverse types of cancer, contributing substantially to the global onus of diseases, death and disability. Thus, sedentary lifestyle is also recognized as one of the most modifiable risk factors for those pathologies [1–2].

Previous studies have reported the prevalence of sedentary lifestyle in several countries and have discussed its health impact on society [3–4]. In 2002, European countries presented a general sedentariness rate of 31%, with the lowest rates found in the Netherlands and Denmark, 19.3 and 22.3% respectively, and the highest rates in France and Belgium, 43.1 and 39.8% respectively [3]. Spain, with a current estimated total population of 46.23 million of persons [5], presented a prevalence of sedentary lifestyle of 31.2% in adult population, according to that data from 2002 [3].

In light of the changes in social behavior during the last few decades, which reflect the worrying increasing prevalence of cardiovascular risk factors and type 2 diabetes, new reports containing updated data are essential. The aim of this study was to analyze a current and detailed characterization of PA in Spain though a nationwide, population-based study to offer and to describe its relationship with age, sex, diabetes status, and other cardiovascular risk factors.

## Materials and Methods

The study was approved by the Ethics and Clinical Investigation Committee of Carlos Haya Hospital according with Declaration of Helsinki, and written informed consent was obtained from all participants [6].

### Population

The Di@bet.es Study was a national, cross-sectional, population-based survey conducted in Spain during 2009–2010 to examine the prevalence of diabetes and impaired glucose regulation, and of other cardiovascular risk factors, including physical inactivity [6]. A cluster (n = 100 Primary Care Centers) sampling design was used to select adult participants, forming a representative random sample of the Spanish population. From the initial 5,072 individuals, 4991 completed the Short Form of the International Physical Activity Questionnaire (SF-IPAQ) [7]. Eighty-one questionnaires were incomplete and were considered invalid for the study.

Participants were invited to attend a one-time appointment at their primary health care center. Information was collected using a structured, interview-administered questionnaire followed by a physical examination. (Full questionnaire in original language [S1 File, Diabetes Study Questionnaire in original Spanish-language] and its translation to English [S2 File, Diabetes Study Questionnaire, English translation] are available as Supporting Information). Age, sex, education level, smoking habits, level of adherence to a Mediterranean diet, and personal history of hypertension and dyslipidemia were recorded [8–10]. Weight, height and waist and hip circumferences were directly measured; blood pressure was recorded; and body mass index (BMI) was calculated [6]. Central obesity was defined as waist to hip ratio greater than 0.85 for women and 1 for men. After the interview, a fasting blood sample was obtained and a standard oral glucose tolerance test was performed in individuals without known diabetes (KDM) to identify those subjects with unknown diabetes (UKDM) and PREDM (prediabetes: impaired glucose tolerance and/or impaired fasting glucose) [11]. Participants who refused the OGTT (n = 1,429) were classified according to their fasting glucose levels. Atherogenic dyslipidemia was defined as triglycerides > 150mg/dl and HDLc < 40 mg/dl for men or HDLc < 45 mg/dl for women. Homeostatic model assessment (HOMA-IR) was calculated [12].

Physical activity (PA) was estimated using the Short Form of the International Physical Activity Questionnaire (SF-IPAQ), in which individuals report the number of days and the duration of the vigorous, moderate, and walking activities during the previous week [4]. These data were quantified, and an estimated MET (Metabolic Equivalent of Task) was attributed to each activity [4]. According to IPAQ score, each individual was assigned to one of three categories: high, moderate, and low PA [7]. Those individuals who did not reach a minimum of minutes and/or days per week of vigorous, moderate or walking activities met the criteria for low PA levels and were thus considered sedentary individuals [3,4,7]. Conversely, those individuals meeting criteria for high or moderate PA categories were identified as active individuals.

### Statistical analysis

Data are presented as median and interquartile range and number of individuals and percentage, unless otherwise indicated. To estimate prevalence of sedentariness in Spain, standardized rates (x100 pop.) of low PA category (from IPAQ) were calculated using the direct method. The age and sex structure of the Spanish population (National Institute of Statistic [INE] 2010) was used as the standard population [5]. Anthropometric, clinical, and laboratory differences across three glucose metabolism categories (KDM, PREDM/UKDM and normal glucose regulation [NORMAL]) or between sedentary vs. active individuals were evaluated by the chi-squared test (for categorical), Wilcoxon or Kruskal-Wallis test (for continuous non-normally distributed), or student’s t-test or one-way ANOVA (for continuous normally distributed), respectively. Multiple logistic regression models or adjusted general linear models (PROC GLM in SAS) were used to investigate factors associated with physical activity (dependent) variables after adjustment for confounders (age, sex, BMI, glucose metabolism categories etc). These tests were also used to evaluate age*sex interactions (with or without additional BMI adjustment) in the association between age, sex, and PA. Finally, logistic multiple stepwise (level of entry p < 0.15) regression analysis was used to investigate variables independently associated with sedentary lifestyle. In the Di@bet.es Study [6] the sample size was calculated to investigate the prevalence of diabetes in Spain. The significance level was defined as p ≤ 0.05. Analyses were performed with SAS software, v.9.2 (SAS Institute Inc., USA).

## Results

### Prevalence of sedentary lifestyle, or low physical activity level

**Fig 1:**
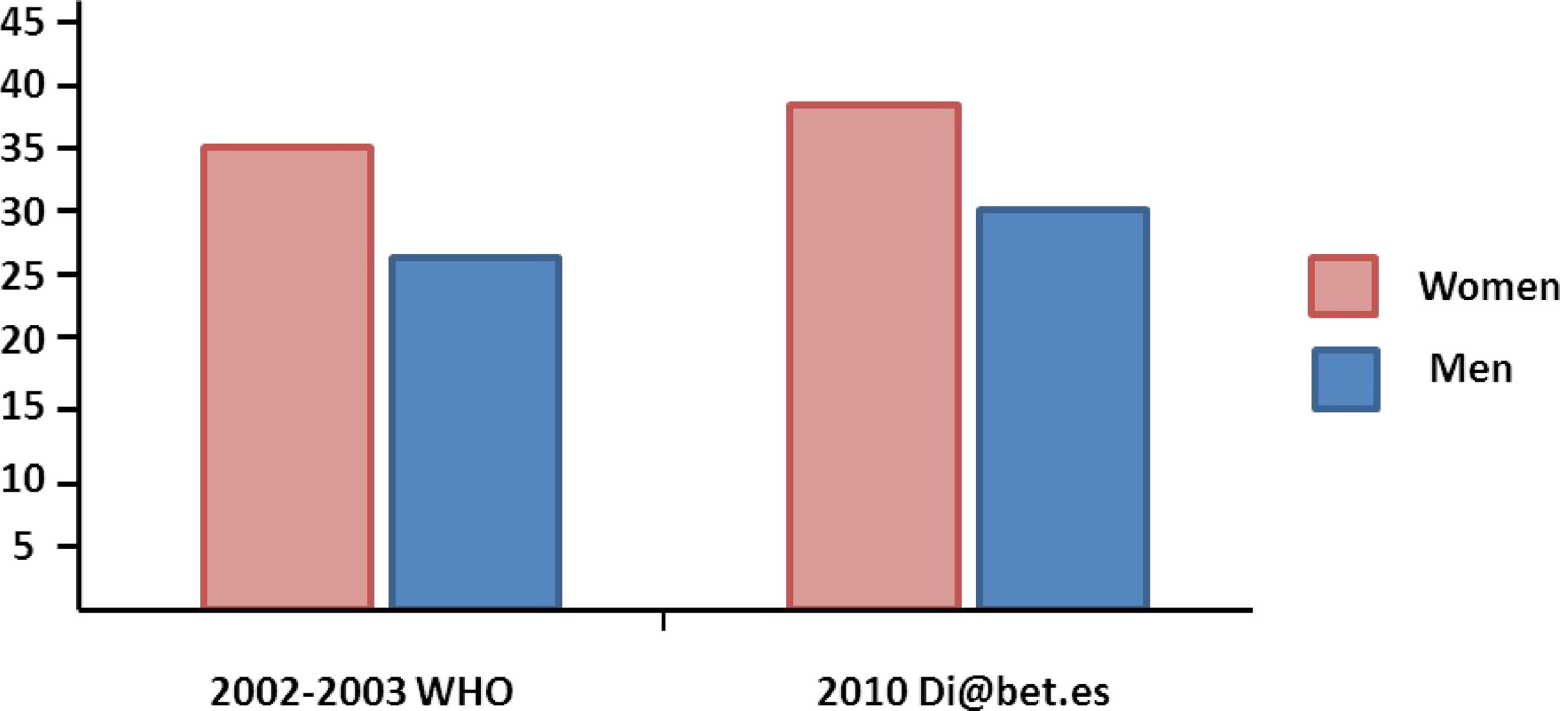
Prevalence of sedentariness, or low physical activity, in Spain. Prevalence was adjusted to the age and sex structure of the Spanish population. *Data from the 2002–2003 report by Sjöström M et al in 2006 [3].

Data from 4991 individuals were analyzed. Mean ± SD age was 50 ± 17 years, 57% women, and mean BMI was 28.1 ± 5 kg/m^2^. The overall age-and-sex standardized prevalence of sedentary lifestyle in Spain was 35.7% (IC 34.3–37). Females (39% [IC 37.1–40.8]) were more sedentary than males (32.3% [IC 30.3–34.3]) (p < 0.0001) (Fig 1).

**Fig 2:**
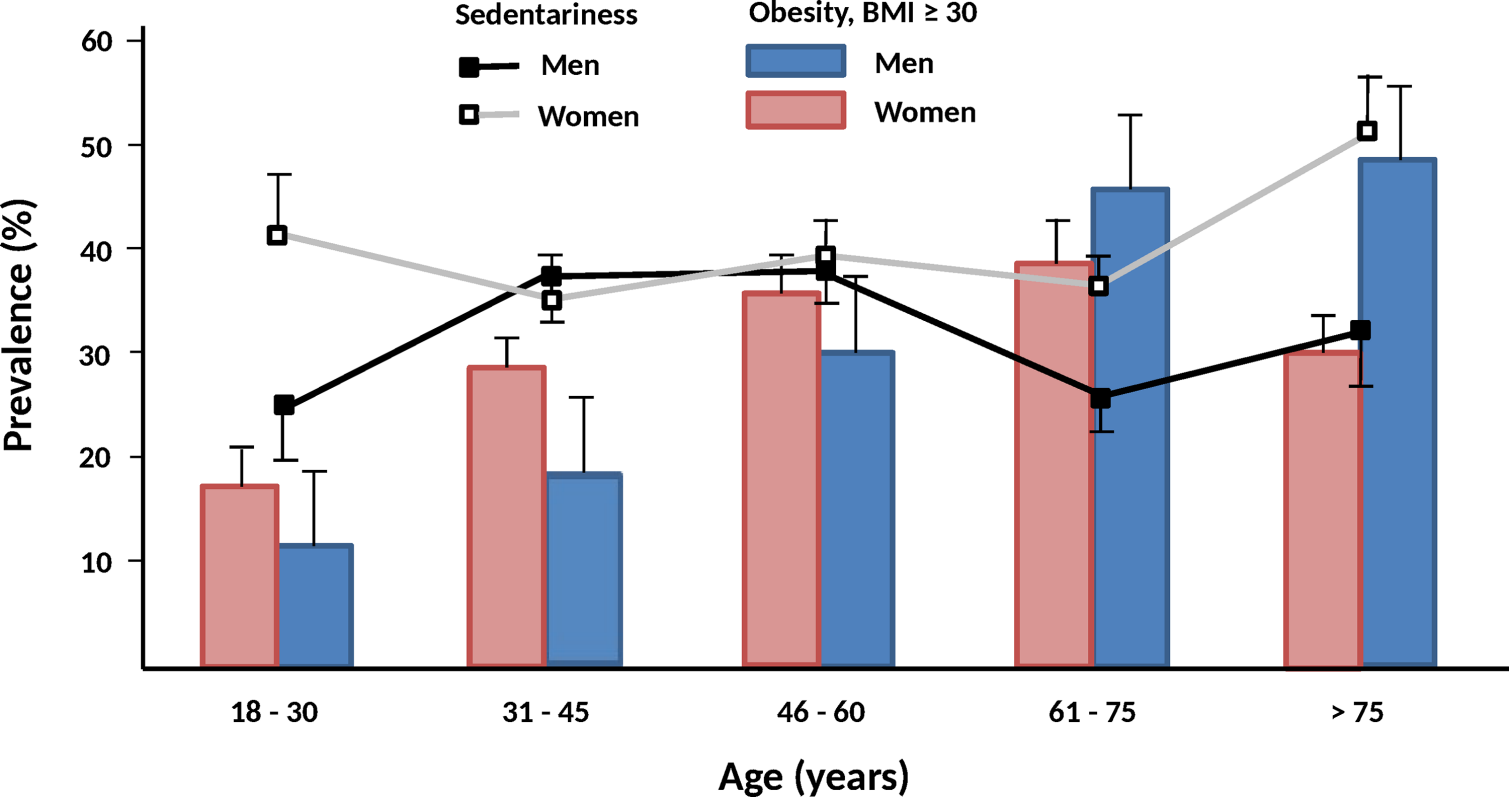
Prevalence of sedentariness and obesity in men and women in Spain. Data adjusted to the age and sex structure of the Spanish population (INE 2016) [5]. BMI, body mass index.

Although age was not univariately associated (p = 0.48) with sedentary lifestyle, there was a positive age*sex interaction (p = 0.0029) in the association between age and PA, with women being especially sedentary at early (18–30 years) and advanced (> 60 years) ages as compared to men (Fig 2).

This interaction was attenuated (p = 0.07) after adjusting for BMI, a potential confounder in the association between physical activity, age, and sex (Fig 2). Sex differences in physical activity were further explored in our population and showed in Table 1.

**Table 1:** Physical activity in women and men.

	Women						Men					
Age group (years)	18-30(n = 334)	31-45(n = 810)	46–60 (n = 823)	61–75 (n = 613)	>75 (n = 284)	All(n = 2864)	18–30 (n = 260)	31–45 (n = 594)	46–60 (n = 542)	61–75 (n = 519)	>75(n = 212)	All(n = 2127)
No physical activity at all	32 (10)	61 (8)	66 (8)	56 (9)	52 (18)	264 (9)	8 (3)	43 (7)	49 (9)	17 (3)	17 (8)	134 (6) ^†^
**Walking**												
Walking activity at least 10 min once a week	287 (86)	719 (89)	739 (90)	553 (90)	230 (81)	2528 (88)	219 (84)	506 (85)	470 (87)	490 (94)	192 (91)	1877 (88)
MET-min/week, median (p25^th^ -p75^t^h) ^a^	396(231–792)	594(297–1188)	594(264–1040)	594(330–1386)	594(297–1040)	594(297–1040)	495(248–1040)	495(248–990)	627(297–1188)	990(462–1386)	990(478–1386)	693(330–1386) ^†^
Walking time, min/week, median (p25^th^ -p75^t^h) ^a^	120(70–240)	180(90–360)	180(80–315)	180(100–420)	180(90–315)	180(90–315)	150(75–315)	150(75–300)	190(90–360)	300(140–420)	300(145–420)	210(100–420) ^†^
**Moderate and Vigorous Physical Activity (MVPA)**												
MVPA at least once a week	146 (44)	365 (45)	319 (39)	200 (33)	61 (22)	1091 (38)	202 (78)	320 (54)	232 (43)	204 (39)	63 (30)	1021 (48) ^†^
MET-min/ week, median (p25^th^ -p75^t^h) ^b^	840(480–1920)	960(480–1680)	960(480–1920)	720(380–1680)	720(240–1680)	960(480–1800)	1440(720–3000)	1200(480–2880)	1440(480–2580)	960(480–1920)	960(320–2400)	1200(480–2400) ^†^
MVPA time, min/week, median (p25^th^ -p75^t^h) ^b^	165(60–300)	180(90–360)	210(120–480)	180(90–420)	180(60–420)	180(90–360)	240(100–480)	180(85–412)	240(108–450)	210(113–420)	210(70–540)	210(90–435) ^†^
**Overall Physical activity**												
**Total MET-min/week, median (p25**^**th**^**-p75**^**t**^**h)**	693(248–1536)	834(330–1695)	693(297–1533)	693(297–1485)	498(148–1188)	693(297–1506)	1542(693–3329)	988(346–2430)	883(346–2141)	1386(594–2190)	1093(404–2079)	1150(446–2400) ^†^
Total PA min/week, median (p25^th^ -p75^t^h)	180(70–380)	212(100–420)	210(90–420)	210(90–430)	150(45–360)	210(80–420)	330(140–660)	210(90–540)	227(100–560)	360(170–630)	300(120–630)	290(120–600) ^†^
> 1000 MET-min/week of physical activity, n (%) ^**c**^	126 (38)	362 (45)	328 (40)	240 (39)	82 (29)	1138 (40)	169 (65)	288 (48)	249 (46)	298 (57)	110 (52)	1114 (52) ^†^

Data are number of individuals and percentage or median and interquartile range

^a^ Data from individuals walking at least 10 minutes once a week

^b^ Data from individuals performing MVPA at least once a week

^c^ Health & Human Services 2008 recommendation [13]

^†^ p<0.001 for sex differences

There were a higher proportion of women performing no PA at all (neither walking nor moderate/intense PA) as compared to men. Furthermore, women spent less time (minutes/week) exercising, and had lower energy expenditure associated with walking, moderate/vigorous (MVPA), and total physical activities (MET-minutes/week) (Table 1). The proportion of individuals achieving the general recommendation of weekly PA, such as ≥ 1000 MET-minute/week [13], was also lower in women. Finally, similar interaction terms sex*age (all p ≤ 0.05) to the one previously described (Fig 2) for the association between sedentary lifestyle and age were also found for most of the PA variables shown in Table 1, with the exception of total MET-minute/week, MVPA MET-minute/week, MVPA min/week, and proportion of individuals achieving ≥ 1000 MET-minute/week (p = 0.67, p = 0.36, p = 0.23, and p = 0.12, respectively).

### Association of physical activity and anthropometric, laboratory, and clinical variables: univariate analysis

As expected, low PA was associated with most of the traditional and non-traditional cardiovascular risk factors, such us, obesity and waist circumference, hypertension, dyslipidemia, atherogenic dyslipidemia, active smoking, HOMA–IR index, and fasting glucose, triglycerides and HDL-cholesterol levels, adherence to a traditional Mediterranean diet, and educational level (Table 2).

**Table 2:** Subjects’ clinical and laboratory characteristics according to physical activity.

	Physical activity		
	Sedentary (n = 1945)	Active (n = 3046)	p
**Obesity** (BMI ≥ 30 kg/m^2^)	721 (37)	803 (26)	<0.0001
BMI (kg/m2)	28.2 (24.8–31.9)	27.1 (24.2–30.3)	<0.0001
Central obesity (n,%) ^a^	921 (48)	1122 (37)	<0.0001
Waist circumference (cm)	96 (86–106)	93 (83–102)	<0.0001
**Hypertension** (on treatment)	510 (26)	665 (22)	0.0004
On 1 antihypertensive drug (n,%) ^b^	265 (14)	399 (13)	0.5939
On > 1 antihypertensive drugs (n,%)	200 (10)	250 (8)	0.0125
Systolic blood pressure (mm Hg)	130 (116.5–144.3)	130 (117–144.3)	0.6856
Diastolic blood pressure (mm Hg)	76.5 (70–84)	76.5 (69.6–83.5)	0.2345
**Dyslipidemia**	702 (37)	951 (32)	0.0004
On lipid lowering medication (n,%)	278 (14)	397 (13)	0.2045
On statins (n,%)	219 (11)	358 (12)	0.5949
Atherogenic dyslipidemia (n,%) ^c^	200 (11)	224 (8)	0.0003
Total-cholesterol (mg/dL)	196 (169–222)	195 (167–222)	0.4717
HDL-cholesterol (mg/dL)	49 (42–58)	51 (43–60)	0.0026
Triglycerides (mg/dL)	108 (78–148)	99 (73–136)	<0.0001
**Glycemia** (mg/dL)	94 (86–105)	93 (85–103)	0.0057
HOMA-IR	2.03 (1.36–3.21)	1.74 (1.20–2.60)	<0.0001
On diabetes medication (n,%)	176 (9)	233 (8)	0.0788
On insulin treatment (n,%)	40 (2)	40 (1)	0.0414
**Mediterranean diet adherence**(lower tertile) (n,%)	788 (41)	1025 (34)	<0.0001
**Current smoker** (n,%)	540 (28)	745 (25)	0.0089
**University education** (n,%)	279 (14)	507 (17)	0.0301

Data are number of individuals and percentage or median and interquartile range. P-values for group differences are given

^a^ Central obesity: waist / hip ratio ≥ 1 in men, ≥ 0.85 in women

^b^ Missing data for antihypertensive drugs n = 61

^c^ Atherogenic dyslipidemia: triglycerides > 150 mg/dl and HDL-cholesterol < 50 mg/dl for women or < 40 mg/dl for men

### Association of physical activity and glucose metabolism

Age, BMI and sex distribution were different among individuals with different glucose metabolism categories. Therefore, the association between sedentariness and other PA variables, and glucose metabolism (a three-level variable) was explored before and after adjustment for age, sex, and BMI (Table 3).

**Table 3:** Physical activity in carbohydrate metabolism status.

	NORMAL(N = 3771)	PREDM/UKDM (N = 808)	KDM(N = 472)	* p value	^†^ Age-sex-BMIadj p-value
Age (years)	45 (34–58)	61 (51–72)	66 (59–74)	<0.0001	-
Male sex, n (%)	1483 (40)	404 (50)	240 (51)	<0.0001	-
BMI (kg/m^2^)	26.6 (23.8–29.8)	30.3 (27.3–33.4)	30.2 (27.1–33.3)	<0.0001	-
No physical activity at all, n (%)	248 (7)	82 (10)	68 (14)*	<0.0001	<0.0001
**Sedentariness, n (%)** ^c^	1392 (38)	346 (43)	207 (44)	0.0014	0.0160
**Walking**					
Walking activity at least 10 min once a week	3308 (89)	706 (87)	391 (83)	0.0002	<0.0001
MET-min/week, median (p25^th^ -p75^t^h) ^a^	594 (297–1188)	693 (297–1386)	693 (330–1386)	0.0255	0.7927
Total time, min/week,median (p25^th^ -p75^t^h) ^a^	180 (90–360)	210 (90–420)	210 (100–420)	0.0255	0.7926
**Moderate and Vigorous Physical Activity (MVPA)**					
MVPA at least once a week	1703 (46)	274 (34)	135 (29)	<0.0001	0.0258
MET-min/week, median (p25^th^ -p75^t^h) ^b^	960 (480–2320)	960 (480–1920)	960 (400–1920)	0.0929	0.2132
Total time, min/week,median (p25^th^ -p75^t^h) ^b^	180 (90–420)	180 (90–420)	240 (90–360)	0.9965	0.2705
**Total MET-min/week median (p25**^**th**^ **-p75**^**t**^**h)**	932 (358–2020)	718 (297–1615)	693 (231–1628)	<0.0001	0.1544
Total PA min/week,median (p25^th^ -p75^t^h)	230 (100–480)	210 (80–433)	210 (70–450)	0.0014	0.2770
> 1000 MET-min/week of physical activity (%)	1719 (46)	344 (43)	189 (40)	0.0101	0.1771

Data are number of individuals and percentage or median and interquartile range. P-values for groups differences before (*) and after (^†^) adjustment for age, sex, and BMI (body mass index) are given. NORMAL: normal glucose metabolism; PREDM/UKDM: prediabetes and unknown diabetes; KDM: known diabetes

^a^ Data from individuals walking at least 10 minutes once a week

^b^ Data from individuals performing MVPA at least once a week

^c^ No difference between PREDM/UKDM and KDM (p = 0.72)

After adjustment, glucose metabolism was associated with sedentary lifestyle (p = 0.016) and with performing no PA at all (p < 0.0001). Furthermore, although the prevalence of individuals walking at least 10 min/day once a week (p < 0.0001) or performing moderate-and-vigorous PA once a week (p = 0.0258) decreased steadily from NORMAL to PREDM/UKDM to KDM, the total amount of MET-min/week or min/weeks of those who did perform these activities was not different across glucose metabolism categories after adjustment. We found no differences between groups in total physical activity MET-min/week or min/week, or in the percentage of individuals performing > 1000 MET-min/week of PA.

Afterwards, we compared age-sex-and–BMI adjusted PA differences between individuals with KDM and those with PREDM/UKDM. We found that individuals with KDM had a higher probability of not performing PA at all (OR 95 CI: 1.48 [1.03–2.11], p = 0.03), a lower probability of walking at least 10 min/day once a week (OR 95 CI: 0.67 [0.49–0.93], p = 0.02), and a similar chance of performing MVPA once a week (OR 95 CI: 0.87 [0.67–1.12], p = 0.28), compared with those with PREDM/UKDM. Furthermore, the prevalence of sedentariness (p = 0.59), or other PA variables shown in Table 3, was not different between these two groups after age-sex-and-BMI adjustment.

### Multiple regression analysis

Finally, in a multiple logistic regression analysis (Table 4), we found that the variables independently associated with low PA were age, sex, BMI, central obesity, Mediterranean diet adherence, dyslipidemia, hypertension (p = 0.1), smoking habit and HDL-cholesterol and triglycerides concentrations. Insulin use, or treatment with more than one antihypertensive drug, was not independently associated with low PA in these models.

**Table 4:** Variables associated with sedentariness, or low physical activity.

	OR (95% CI)
Age (5 years increment)	0.98 (0.95–0.99)
Male sex	0.63 (0.55–0.73)
BMI (5 units kg/m^2^ increment)	1.23 (1.15–1.31)
Central obesity (WHR ≥1m men; ≥0.85m women)	1.20 (1.04–1.40)
Dyslipidemia	1.16 (1.01–1.32)
Hypertension	1.15 (0.97–1.37)
HDL-cholesterol (5 mg/dl increment)	0.97 (0.94–0.99)
Triglycerides (10 mg/dl increment)	1.01 (1.00–1.02)
Adherence to Mediterranean Diet (2^nd^-3^rd^ tercile vs.1^st^ tercile)	0.74 (0.65–0.84)
Active smoker	1.25 (1.08–1.44)

Data are OR (95% CI) for the association between the variables indicated and physical activity. Multiple logistic regression model (stepwise), level of sig. for entry p = 0.15. Dependent variable: sedentariness, or low physical activity (low vs. moderate/vigorous). Independent variables considered in the model were age, sex, BMI (kg/m^2^), central obesity (waist/hip ratio ≥ 1 in men, ≥ 0.85 in women), carbohydrate metabolism (NORMAL, PREDM/UKDM, KDM), active smoking, hypertension, dyslipidemia, university studies, Mediterranean diet adherence, HDL-cholesterol (mg/dl), triglycerides (mg/dl), systolic and diastolic blood pressures (mmHg), insulin use, and > 1 hypertensive drug use.

## Discussion

This nationwide population-based study provides current information on the prevalence of low PA (sedentariness) and other IPAQ derived variables in Spain. Age-and-sex standardized rate of sedentary lifestyle (low PA) was 35.7% (32.3% for men and 39% for women). These ciphers are higher than previous data from 2002 (Fig 1), when 31.2% of the Spanish population (27.3% for men and 34.9% for women) were sedentary, using the same methodology (SF-IPAQ questionnaire). Therefore, we observed that sedentariness, one of the most important risk factors for several chronic diseases, as obesity, diabetes and cardiovascular disease, is increasing in Spain. Indeed, our data agree with the pessimist projections of a worsened rate of these conditions in the decades to come [14–16]. The criterion of sedentary lifestyle employed by Sjöström and col. [3] and Guthold and col.[4] was the main variable applied in the present study. Other variables, such as ≥ 1000MET-min/week of PA [13] or no PA at all, were also reported here to facilitate the comparison to other studies and guidelines. Regardless of the different indexes presented, all of them show the same tendency of a lack of compliance with the health guidelines of recommended PA.

We observed that the association between age and PA was not linear and was associated with sex and BMI (Fig 2). Younger (< 30 years) and older women (> 60 years) were more sedentary that men in the same age group. Previous studies have also shown lower rates of PA in younger women as compared to men, and these rates become similar with an increase in age [17,18]. However, our multiple regression analysis (Table 4) showed an inverse association between aging and sedentary lifestyle after accounting for many confounders, adding further proof to the complex nature of this relationship.

Other studies also reported association of sedentariness with cardiovascular risk factors. Arteaga and col.[18] demonstrated an inverse association of PA with insulin resistance in young adults. Leisure time PA was inversely associated with BMI, waist circumference and HOMA-IR in a study of the Swedish population [19]. Previous European data [20] also showed an association of sedentary lifestyle and obesity with lower educations levels. In our study, sedentariness was associated with most cardiovascular risk factors (Table 4) including anthropometric, laboratory, clinical, and lifestyle factors (i.e. adherence to a cardiovascular-healthy diet) [21].

We also explored PA across glucose metabolism categories (normal glucose regulation, PREDM/UKDM, and KDM) and found lower PA from normal to PREDM/KDM to KDM glucose metabolism categories (Table 3). Furthermore, to avoid the confounding effect of clinical intervention for diabetes treatment, PA in individuals with KDM was also compared with the closer group (based on age, sex distribution, and BMI), i.e., PREDM/UKDM individuals. No major differences (according to healthy recommendations) between these two groups (Table 3 and Results section) were found. A recent study developed in Spain [22] also analyzed known and unknown diabetes and identified that patients that already knew of their diabetes had more medical advice to practice PA, but in fact, they did the same amount of PA as those with unknown diabetes. Other studies also indicate that adults with diabetes have lower levels of PA than those without diabetes [23,24]. This indicates that those patients with known diabetes perform no more beneficial (sufficient or therapeutic) PA than those at-risk individuals who are not aware of their condition. This finding points to a possible failure in the current strategies for promoting PA, even in individuals with diabetes, who should have received formal indications to increase PA as part of their treatment, and would prompt diabetes health providers to improve PA prescription and/or adherence. On the other hand, Murillo and col. [25] reported that Spanish patients with type 2 diabetes were more likely to perform PA, mainly walking, as compared to European data. Other recent encouraging results were reported in a longitudinal study conducted in the USA of 68,132 women with recently diagnosed diabetes, showing an increase in PA and a reduction in sedentary behavior following the diagnosis of diabetes [26]. Studies such as DPP [27], DPS [28] and Look AHEAD [29] are clear in demonstrating the importance and effectiveness of a PA program for preventing diabetes in persons at risk for (with prediabetes) or with established type 2 diabetes.

The main strength of the study is that the Di@bet.es Study was designed to be representative of the whole national territory, enabling the evaluation of several clinical, biochemical and demographic characteristics of the Spanish population during the period 2009–2010. In addition, the SF-IPAQ is an easy and reproducible tool that has already been validated by different countries and cultures, facilitating the comparison and contrast to other populations. However, this questionnaire also has some limitations. The SF-IPAQ is not as precise as tools like accelerometers [30], particularly in the evaluation of the category of high PA. Furthermore, over-reporting of PA by those interviewed must be considered (discussed in Strath and col. [31] and Felix-Redondo and col. [32]) and, in fact, the prevalence of sedentariness could actually be even higher. Finally, our general study questionnaire did not contemplate specific health conditions that could hamper the practice of PA and are found more frequently in persons with type 2 diabetes and/or obesity, which could explain, in part, the levels of sedentary lifestyle in these groups [33]. Nevertheless, this is a limitation that commonly affects large epidemiological surveys.

## Supporting Information

S1 File Diabetes Study Questionnaire in original Spanish-language. (PDF)

S2 File Diabetes Study Questionnaire, English translation. (PDF)
