# Supplementary material for: Low Physical Activity and Its Association with Diabetes and Other Cardiovascular Risk Factors: A Nationwide, Population-Based Study
Source: PLoS One. 2016 Aug 17;11(8):e0160959. doi: 10.1371/journal.pone.0160959 (PMC4988632; doi:10.1371/journal.pone.0160959)
Supplement: S1 File — (PDF) [file pone.0160959.s001.pdf]

di@bet.es
Estudio Epidemiológico de la Diabetes tipo 2 en España

Pegar etiqueta identificativa

DATOS IDENTIFICATIVOS

CÓDIGO:.....................................FECHA :.............................EQUIPO:..................................

NOMBRE:.. APELLIDOS:……………………………………………………………………………………………

DOMICILIO: ....CP.…..............PUEBLO/ CIUDAD..................................PROVINCIA..................................

TELÉFONO: ....FECHA NACIMIENTO: .... EDAD ACTUAL:................... ☐HOMBRE ☐MUJER

PAÍS DE ORIGEN: ....ETNIA:..ENCUESTADOR: ……………………………………

CRITERIOS DE EXCLUSIÓN

	**Señalar si se cumplen alguno de los criterios:**
1-Reside Ud. en la actualidad en algún hospital, residencia o institución?<br>1☐ SI 2 ☐ NO 9☐ ns, nc	☐ EXCLUIDO
2-¿Está Ud. embarazada?<br>1☐ SI 2☐ NO 9 ☐ ns, nc	☐ EXCLUIDO
3-¿Ha dado Ud. a luz en los últimos 6 meses?<br>1 ☐ SI 2☐ NO 9☐ ns, nc	☐ EXCLUIDO
4-¿Ha sufrido Ud. alguna intervención quirúrgica mayor en el último mes?<br>1 ☐ SI 2☐ NO 9☐ ns, nc	☐ EXCLUIDO
5-¿Ha sufrido Ud. alguna enfermedad grave no relacionada con la diabetes en los últimos 6 meses?<br>1☐ SI 2☐ NO 9☐ ns, nc	☐ EXCLUIDO

Si excluido, pasar a página 4: Diabetes Conocida, HTA y enfermedades cardiovasculares

ESTATUS SOCIAL

6-Estado Civil

1☐ Soltero/a
2☐ Casado/a o en pareja
3☐ Viudo/a
4☐ Separado/a divorciado/a
9☐ ns / nc

7-¿Qué estudios ha realizado usted?

1☐ No sabe leer/escribir
2 ☐ Sin estudios
3 ☐ Estudios primarios (hasta 12 años)
4☐ Estudios secundarios (hasta 16 años)
5☐ Bachiller, formación profesional, (hasta 18 años)
6☐ Universitarios primer ciclo (diplomado)
7☐ Universitarios (licenciado, ingeniero, doctor)
9☐ ns / nc

8-¿Trabaja fuera de casa?

1☐ SI 2☐ NO 9☐ ns / nc

9-Si no trabaja ¿por qué motivo?

1☐Jubilado
2☐ En paro
3 ☐ Baja por enfermedad
4☐ estudiando
5☐ ama de casa
9☐ ns / nc

10-¿Cuál es su ocupación actual o la que ha desempeñado la mayor parte de su vida? Anotar …………………………………………………

1☐Directivos de la Administración Pública y de empresas de 10 o más asalariados.
Profesiones asociadas a titulaciones de 2º y 3er ciclo universitario.
2☐Directivos de empresas con menos de 10 asalariados.
Profesiones asociadas a una titulación de 1er ciclo universitario.
Técnicos Superiores. Artistas y deportistas
3☐ Empleados de tipo administrativo y profesionales de apoyo a la gestión administrativa y financiera.
Trabajadores de los servicios personales y de seguridad.
Trabajadores por cuenta propia.
Supervisores de trabajadores manuales.
4 ☐Trabajadores manuales cualificados.
5 ☐Trabajadores manuales semicualificados.
6☐Trabajadores no cualificados.
7☐ Otros (clero, militares,...).

HÁBITO TABÁQUICO

11-¿Fuma Ud?

1☐Si 2☐No 9☐ns/nc

12-¿Cuántos cigarrillos fuma? ..../día

13-¿A qué edad empezó a fumar?...................

14-¿Ha cambiado la cantidad de tabaco fumado en los últimos 6 meses?

1☐SI, fumo más 3☐SI, fumo menos

2☐NO 9☐ns / nc

<u>SI NO FUMA ACTUALMENTE</u>

15-¿Ha fumado Ud. más de 1 cig./día en alguna época de su vida?

1☐Si 2☐No 9 ☐ns/nc

16-¿Cuánto solía fumar?................

17-¿A qué edad dejó de fumar?..........

18-¿A qué edad empezó a fumar?..........

ACTIVIDAD FÍSICA

19-¿Cuántas horas suele dormir? .... horas/día

20- ¿Cuál de estas posibilidades describe mejor su actividad principal en el centro de trabajo, centro de enseñanza, hogar (labores domésticas), etc.?

1 ☐ Sentado la mayor parte de la jornada

2 ☐ De pie la mayor parte de la jornada sin efectuar grandes desplazamientos o esfuerzos

3 ☐ Caminando, llevando algún peso, efectuando desplazamientos frecuentes que no requieran gran esfuerzo físico

4 ☐ Realizando tareas que requieren gran esfuerzo físico

21- En su tiempo libre ¿Realiza habitualmente ejercicio físico al menos una vez por semana?

1☐Si 2☐No 9☐ns /nc

Si la respuesta es sí:

22- ¿Cuántos días por semana? ....días.

23- Los días que hace ejercicio ¿Cuántas hora practica? .... horas.

24- ¿Qué tipo de ejercicio practica**? ....**

ANTECEDENTES DE DIABETES

25- ¿Alguna vez le ha dicho un médico que tuviera Ud. diabetes? (azúcar en la sangre) 1☐Si 2☐No 9☐ns/nc

26- ¿Qué edad tenía? ....años

27- ¿Alguno de sus familiares (padres, hermanos, hijos, abuelos) **tienen diabetes?**
1☐Si 2☐No 9☐ns/nc

Especificar cuantos:

28- Abuelos: ....

29- Padres: ....

30- Tíos: ....

31- Hermanos: ....

32- Hijos**:** ....

33- ¿Toma alguna pastilla para la diabetes?
1☐Si 2☐No 9☐ns/nc

34- ¿Cuánto tiempo ha estado tomándolas? ....años

35- ¿Se inyecta Ud. insulina?
1☐Si 2☐No 9☐ns/nc

36- ¿Cuánto tiempo lleva poniéndose insulina? ....años

37- ¿Le ha puesto a dieta el médico por su diabetes?
1☐Si 2☐No 9☐ns/nc

38- ¿Cuanto tiempo hace que sigue la dieta? ....años
(Si no la sigue poner 77)

ANTECEDENTES DE HIPERTENSIÓN ARTERIAL

39- ¿Le ha dicho alguna vez un médico que tuviera la tensión arterial alta? 1☐Si 2☐ No 9☐ns/nc

40- ¿Qué edad tenía cuando le dijeron que tenía hipertensión? ....años.

41- ¿Toma alguna medicación para la HTA? 1☐Si 2☐ No 9☐ ns/nc

42- ¿Cuánto tiempo ha estado tomándolas? ....años

43- ¿La ha tomado en las últimas 24 horas? 1☐ Si 2☐No 9☐ns/nc

ENFERMEDADES CARDIOVASCULARES Y OTRAS

44- ¿Le ha dicho alguna vez un médico que tuviera el colesterol alto?

1☐Si 2☐No 9☐ns/nc

45- ¿Le ha dicho un médico que haya sufrido un ataque cardíaco, angina de pecho o infarto de miocardio?

1☐Si 2☐No 9☐ns/nc

46- ¿Le ha dicho un médico que haya tenido una trombosis cerebral, embolia cerebral, hemorragia cerebral o parálisis cerebral?

1☐Si 2☐No 9☐ns/nc

47- ¿Le ha dicho alguna vez un médico que tuviera vasculopatía periférica (úlceras en el pie)?

1☐Si 2☐No 9☐ns/nc

48- ¿Le ha dicho un médico que padeciera alguna enfermedad crónica (bronquitis, asma, reumatismo, tumores etc?

1☐Si 2☐No 9☐ns/nc **49- Especificar:** ....

**FÁRMACOS que toma habitualmente, incluyendo vitaminas, analgésicos, tranquilizantes, ... etc. (no incluir productos de herbolario)**	
50..	55..
51..	56..
52..	57..
53..	58..
54..	59..

HISTORIA GINECOLÓGICA

60-¿Cuántos partos ha tenido? .... **Pesos de los niños al nacer**: ....kg, ...kg, ...kg, ...kg, ...kg, ...kg,

61- ¿Ha tenido abortos?, ¿Cuántos? ....

62- ¿Le ha dicho alguna vez el médico que tuviera diabetes gestacional?

1☐Si 2☐No 9☐ns/nc **63- ¿En cuantos embarazos**? ....

64- ¿Qué edad tenía cuando tuvo la regla por primera vez?:.......... años

65- ¿Utiliza anticonceptivos hormonales? (orales o parches)

1☐ Si 2☐No 9☐ns/nc ¿Cuál? ....

66- ¿Cuánto tiempo lleva tomándolos? (aunque haya cambiado de compuesto varias veces) .... años

67-¿Cuándo ha sido la fecha de su última regla? ....

68- Si **lleva más de 6 meses de amenorrea (falta de regla) ¿Cuál ha sido la causa?**

1☐Natural 2☐Quirúrgica 9☐ns/nc

69-¿Está a tratamiento por la menopausia?

1☐ Si 2☐ No 9☐ns/nc **70- Especificar:** ....

71- ¿Cuánto tiempo lleva a tratamiento? (aunque haya cambiado de compuesto varias veces) .... años

CAMBIOS DE PESO

72- ¿Recuerda cuánto pesaba a los 18 años? ....

73- ¿Pesa más ahora que cuando tenía 18 años?
1☐Si 2☐ No, peso igual 3☐No, peso menos 9☐ns/nc

74- ¿Ha perdido peso en el último año?
1☐Si 2☐No 9☐ns/nc

75- ¿Cuántos kg ha perdido? ....kg

76- ¿Ha sido involuntariamente?
1☐Si 2☐No 9☐ns/nc

77- ¿Ha ganado peso en el último año?
1☐Si 2☐No 9☐ns/nc

78- ¿Cuántos kg ha ganado? ....kg

79- ¿A Ud le gustaría...?
1☐Ganar peso 2☐Perder peso 3☐Está conforme con su peso

80- En su opinión Ud. se encuentra:
1☐Delgado 2☐Normal
3☐sobrepeso 4☐Obeso 9☐ns/nc

ANTROPOMETRÍA Y TENSIÓN

81- Peso: .... kg

82- Talla: ....cm

83- Cintura: ....cm

84- Cadera: ....cm

85- Tensión sistólica 1: ....mmHg

86- Tensión diastólica 1: ....mmHg

87- Tensión sistólica 2: ....mmHg

88- Tensión diastólica 2: ....mmHg

Si no concuerdan (diferencia >5mmHg), realizar una 3ª medida:

89- Tensión sistólica 3: ....mmHg

90- Tensión diastólica 3: ....mmHg

91- Si alguna medida no puede realizarse indicar cual y el motivo:
....
....
....

OBSERVACIONES

HÁBITOS ALIMENTARIOS

92- ¿Ha cambiado Ud. la cantidad que solía comer en los últimos 6 meses?
1☐Si, como más   3☐Si, como menos
2☐No, como igual   9☐ns/nc

93- ¿Ha cambiado el tipo de alimento en su dieta durante los últimos 6 meses?
1☐Si   2☐No   9☐ns/nc

94- Especificar el cambio: …………………………..............………………

95- Si la respuesta ha sido sí, ¿Fue por recomendación de algún médico o dietista?
1☐Si   2☐No   9☐ns/nc

96- Si está a dieta, que tipo de dieta: ....
....

97- ¿Cuántas veces come fuera de casa a la semana?
1 ☐ a diario,   4 ☐ uno por semana
2 ☐ de 6 a 4 por semana   5 ☐ de 3 a 2 veces al mes
3 ☐ de 3 a 2 por semana   6 ☐ una vez al mes
7 ☐ casi nunca

98- ¿Toma algún tipo de suplemento vitamínico?
1 ☐ Si   2 ☐ No   9 ☐ ns / nc, **Especificar** ....

99- ¿Toma usted sacarina o algún tipo de edulcorante sin calorías?
☐ 1☐Si   2☐No   3☐ns/nc   100- Especificar ....

100.B- ¿Toma fructosa habitualmente?
1☐Si   2☐No   3☐ns/nc

100.C-¿Qué tipo de sal toma?
1☐Yodada   2☐Marina   3☐Otros   4☐ns/nc

101- ¿Cuántas comidas suele hacer en un día normal?
1☐menos de 3   3☐4 ó 5
2☐3   4☐más de 5

102- ¿Picotea entre horas?
1☐Si   2☐No   3☐A veces   9☐ns/nc

103- ¿Come mientras ve la TV?
1☐Si   2☐No   3☐A veces   9☐ns/nc

104- ¿Qué come mientras ve la TV?
1☐Las comidas normales
2☐Patatas frita, frutos secos, "chucherías"
3☐Ambas cosas

Poner una cruz en el recuadro correspondiente, según con qué frecuencia consuma cada tipo de alimento:

		AL DÍA					A LA SEMANA			AL MES		NUNCA O CASI NUNCA
		5 ó mas veces	4 veces	3 veces	2 veces	1 vez	4-6 veces	2-3 veces	1 vez	2-3 veces	una vez	
105-	Cereales de desayuno											
106-	Cereales de desayuno integrales											
107-	Pan blanco											
108-	Pan integral											
109-	Arroz, pasta italiana, cous-cous											
110-	Arroz, pasta italiana cous-cous integrales											
111-	Azúcar, miel, caramelos, mermeladas											
112-	Chocolate											
113-	Dulces, pasteles, Magdalenas, galletas, donuts, bollos											

	Al día					A la semana			Al mes		NUNCA o casi nunca
	5 ó mas veces	4 veces	3 veces	2 veces	1 vez	4 - 6 veces	2 - 3 veces	1 vez	2 - 3 veces	1 vez	
**114- Huevos**											
**115- Leche, yogurt, queso**											
**116- Batidos envasados, flan natillas, helados**											
**117- Mantequilla, nata, manteca**											
**118- Margarina**											
**119- Mayonesa**											
**120- Frituras caseras**											
**121- Frituras precocinadas/congeladas**											
**122- Otros platos precocinadas (incluyendo pizza)**											

Poner una cruz en el recuadro correspondiente, según con qué frecuencia consuma cada tipo de alimento.

	Al día					A la semana			Al mes		NUNCA o casi nunca
	5 ó mas veces	4 veces	3 veces	2 veces	1 vez	4 - 6 veces	2 - 3 veces	1 vez	2 - 3 veces	1 vez	
**123- Ensaladas** (verduras crudas)											
**124- Menestra o acompañamiento de verdura ✳**											
**125- Verdura en guisos ✳✳**											
**126- Legumbres**											
**127- Patata**, guisada, cocida o frita en casa, NO patatas fritas de bolsa.											
**128- Fruta/ zumos naturales**											
**129- Frutos secos**											
**130- Aperitivos** de bolsa tipo patatas, gusanitos,... etc											
**131- Pescado/marisco frescos**											
**132- Conservas de pescado/marisco**											
**133- Carne, cerdo, ternera, pollo...**											
**134- Embutidos**											

✳Menestra o acompañamiento = verdura u hortaliza que se toman como tales, como plato principal o acompañando al 2º plato, pero no las que se utilicen para preparar guisos, sofritos... etc.

✳✳Verdura en guisos = verdura que acompañe al cocinado (lentejas, estofados, sofritos, etc) y que supone una cantidad pequeña dentro de cada plato.

¿Qué tipo de aceite utiliza habitualmente? (Aquí se pueden marcar varias cruces por ítem)

	**1-Aceite de oliva**	**2-Girasol**	**3-Girasol alto oleico**	**4-Otros**
135- para aliñar (ensaladas, etc)				
136- para guisar				
137- para freír				
138-para mayonesa				

BEBIDAS ALCOHÓLICAS

	**Al Día**					**A la Semana**			**Al Mes**		**Nunca o** casi nunca
	**5 ó mas** veces	**4** veces	**3** veces	**2** veces	**1** vez	**4 - 6** veces	**2 - 3** veces	**1** vez	**2 - 3** veces	**1** vez	
139- Vino tinto											
140- Vino blanco, cava											
141-Vino rosado											
142- Vino dulce											
143- cerveza											
143b-Sidra											
144- licores destilados											

145- ¿Cuándo toma bebidas alcohólicas?
1☐Sólo con las comidas 3☐Ambas
2☐Sólo fuera de las comidas 9☐ns/nc

146- Frecuencia
1☐A diario o casi, 3☐ocasionalmente
2☐Los fines de semana 4☐nunca, 9☐ns/nc

BEBIDAS NO ALCOHÓLICAS

	Al Día					A la Semana			Al mes		Nunca o casi nunca
	**5 ó mas** veces	**4** veces	**3** veces	**2** veces	**1** vez	**4 - 6** veces	**2 - 3** veces	**1** vez	**2 - 3** veces	**1** vez	
147- Cerveza sin alcohol											
148- Refrescos azucarados											
149- Refrescos light											
150- Zumos envasados											
151- Refrescos con cafeína											
152- Café expreso (de máquina)											
153- Café filtrado											
154- Café instantáneo											
155- Café descafeinado (de máquina)											
156- Café descafeinado instantáneo											
157- Té (especificar)											
158- Otras infusiones (especificar)											

159- ¿El cocinero está presente? 1☐Si 2☐No 9☐ns/nc

NOTAS DEL ENTREVISTADOR:

160- Interés: 1☐mucho 2 ☐Medio 3 ☐Poco

161- Precisión en las respuestas: 1 ☐Bien 2 ☐Regular 3 ☐Mal

ENCUESTA DE CALIDAD DE VIDA (Cuestionario SF-12)

Explicar al sujeto que las preguntas que siguen se refieren a lo que él piensa sobre su salud, que sus respuestas nos permitirán saber cómo se encuentra y hasta que punto es capaz de hacer sus actividades habituales. Explicarle que si no está seguro de cómo responder a una pregunta debe contestar lo que le parezca más cierto.

162-En general, usted diría que su salud es:

1☐Excelente 2☐Muy buena 3☐Buena 4☐Regular 5☐Mala

Leer al sujeto: *Las siguientes preguntas se refieren a actividades o cosas que usted podría hacer en un día normal. Su salud actual ¿le limita para hacer esas actividades o cosas? Si es así ¿cuánto?*

163-Esfuerzos moderados, como mover una mesa, pasar la aspiradora o caminar una hora

1-☐ Me limita mucho 2- ☐Si, me limita un poco 3-☐No, no me limita nada

164-Subir varios pisos por la escalera

1-☐ Me limita mucho 2-☐ Si, me limita un poco 3-☐No, no me limita nada

Leer al sujeto: *Durante la últimas cuatro semanas ¿ha tenido alguno de los siguientes problemas en su trabajo o en sus actividades cotidianas a causa de su salud física?*

165- ¿Hizo menos de lo que hubiera querido hacer?

1☐Si 2☐No

166- ¿Tuvo que dejar de hacer algunas tareas en su trabajo o en sus actividades cotidianas? 1☐Si 2☐No

Leer al sujeto: *Durante la últimas cuatro semanas ¿ha tenido alguno de los siguientes problemas en su trabajo o en sus actividades cotidianas a causa de algún problema emocional (como estar triste, deprimido o nervioso)?*

167- ¿Hizo menos de lo que hubiera querido hacer por algún problema emocional?

1☐Si 2☐No

168- ¿No hizo su trabajo o en sus actividades cotidianas tan cuidadosamente como de costumbre, por algún problema emocional?

1☐Si 2☐No

169- Durante las cuatro últimas semanas, ¿ha tenido algún dolor? ☐ Sí ☐ No

169.B-¿hasta qué punto el dolor le ha dificultado su trabajo habitual (incluido el trabajo fuera de casa y las tareas domésticas)?

1☐Nada 2☐Un poco 3☐Regular 4☐Bastante 5☐Mucho

Leer al sujeto: *Las preguntas que siguen se refieren a cómo se ha sentido y cómo le han ido las cosas durante las cuatro últimas semanas. En cada pregunta responda lo que se parezca más a cómo se ha sentido usted*

Durante las cuatro últimas semanas ¿cuanto tiempo...

170- se sintió calmado y tranquilo?

1☐Siempre 2☐Casi siempre 3☐Muchas veces 4☐Algunas veces 5☐Sólo alguna vez 6☐Nunca

171- tuvo mucha energía?

1☐Siempre 2☐Casi siempre 3☐Muchas veces 4☐Algunas veces 5☐Sólo alguna vez 6☐Nunca

172- se sintió desanimado y triste?

1☐Siempre 2☐Casi siempre 3☐Muchas veces 4☐Algunas veces 5☐ Sólo alguna vez 6☐Nunca

---

173- Durante las últimas cuatro semanas ¿con qué frecuencia la salud física o los problemas emocionales le han dificultado sus actividades sociales (como visitar a los amigos y familiares?

1☐Siempre 2☐Casi siempre 3☐Muchas veces 4☐Algunas veces 5☐Sólo alguna vez 6☐Nunca

ENCUESTA DE ACTIVIDAD FÍSICA (IPAQ)

Lea al sujeto: *Ahora le voy a preguntar acerca del tiempo que Usted fue físicamente activo(a) en los últimos 7 días. Por favor responda cada pregunta aún si Usted no se considera una persona activa. Piense acerca de las actividades que Usted hace en su trabajo, como parte del trabajo en el jardín y en la casa, para ir de un sitio a otro, y en su tiempo libre de descanso, ejercicio o deporte.*

Lea al sujeto : *Ahora, piense acerca de todas las actividades vigorosas que requieren un esfuerzo físico fuerte que Usted hizo en los últimos 7 días. Actividades vigorosas son las que hacen respirar mucho más fuerte que lo normal y pueden incluir el levantamiento de objetos pesados, excavar, aeróbicos, o pedalear rápido en bicicleta. Piense solamente en esas actividades que Usted hizo por lo menos 10 minutos continuos.*

174- Durante los últimos 7 días ¿Cuantos días hizo Usted actividades físicas vigorosas?

_____ Días por semana [VDAY; Rango: 0 - 7, 8 ,9]
8. No sabe /no está seguro (a)
9. Rehúsa contestar

Clarificación por parte del entrevistador: *Piense solamente en esas actividades físicas que usted hace por lo menos 10 minutos continuos*
Nota para el entrevistador: *Si la persona entrevistada responde cero*, rehúsa o no sabe, pase a la pregunta 176

175- ¿Cuánto tiempo en total usualmente le toma realizar actividades físicas vigorosas en los días que las realiza?

__ __ Horas por día [VDHRS; Rango: 0-16]
__ __ __ Minutos por día [VDMIN; Rango: 0-960, 998, 999]
998. No sabe /no está seguro(a)
999. Rehúsa contestar

Clarificación por parte del entrevistador: *Piense solamente en esas actividades físicas que usted hace por lo menos 10 minutos continuos*
Nota para el entrevistador: Se está buscando un tiempo promedio por día. Si la persona entrevistada no puede contestar porque la cantidad de tiempo varia día a día, pregunte: "*¿Cuanto tiempo en total le dedicó usted en los últimos 7 días a actividades físicas vigorosas?*"

__ __ Horas por semana [VWHRS; Rango: 0-112]
__ __ __ __ Minutes per week [VWMIN; Rango: 0-6720, 9998, 9999]
9998. No sabe /no está seguro(a)
9999. Rehúsa contestar

Lea al sujeto: *Ahora piense en actividades que requieren esfuerzo físico moderado y que Usted hizo en los últimos 7 días. Actividades físicas moderadas son las que hacen respirar algo más fuerte que lo normal e incluyen cargar cosas ligeras, montar en bicicleta a paso regular, o juego de dobles en tenis. No incluya caminar. Otra vez piense únicamente en aquellas actividades físicas que Usted hizo por lo menos 10 minutos continuos.*

176- Durante los últimos 7 días, cuántos días hizo Usted actividades físicas moderadas?

____ Días por semana [MDAY; Rango: 0-7, 8, 9]
8. No sabe /no está seguro(a)
9. Rehúsa contestar

Clarificación por parte del entrevistador: *Piense solamente en esas actividades físicas que usted hace por lo menos 10 minutos continuos*
Nota para el entrevistador: Si la persona entrevistada responde cero, rehúsa o no sabe, pase a la pregunta 178

177- ¿Cuánto tiempo en total usualmente le dedicó en uno de esos días que hizo actividades físicas moderadas?

__ __ Horas por día [MDHRS; Rango: 0-16]
__ __ __ Minutos por día [MDMIN; Rango: 0-960, 998, 999]
998. No sabe /no está seguro(a)
999. Rehúsa contestar

Clarificación por parte del entrevistador: *Piense solamente en esas actividades físicas que usted hace por lo menos 10 minutos continuos*
Nota para el entrevistador: Se necesita un promedio de tiempo al día de uno de los días en los cuales Usted hizo actividad física moderada. Si la persona entrevistada no puede contestar porque la cantidad de tiempo varia día a día, o incluye tiempo dedicado en diferentes trabajos, pregunte: *¿Cuánto tiempo en total le dedicó Usted en **los últimos 7 días** a hacer actividades físicas moderadas?"*

__ __ __ Horas por semana [MWHRS; Rango: 0-112]
__ __ __ __ Minutos por semana [MWMIN; Rango: 0-6720, 9998, 9999]
9998. No sabe /no está seguro(a)
9999. Rehúsa contestar

Lea al sujeto: *Ahora piense en el tiempo que Usted le dedicó a caminar en los últimos 7 días. Esto incluye caminar en el trabajo y en la casa, caminar para para ir de un sitio a otro, y cualquier otra caminata que Usted haya hecho meramente por recreación, deporte, ejercicio o placer.*

178- ¿Durante los últimos 7 días, cuántos días caminó Usted por lo menos 10 minutos seguidos?

____ Días por semana [WDAY; Rango: 0-7, 8, 9]
8. No sabe /no está seguro(a)
9. Rehúsa contestar

Clarificación por parte del entrevistador: *Piense solamente acerca de la caminata que Usted da por lo menos por 10 minutos seguidos.*
Nota para el entrevistador: Si la persona entrevistada responde cero, rehúsa o no sabe, pase a la pregunta 180

179- ¿Cuánto tiempo en total pasó generalmente caminado en uno de esos días?

__ __ Horas por día [WDHRS; Rango: 0-16]
__ __ __ Minutos por día [WDMIN; Rango: 0-960, 998, 999]
998. No sabe /no está seguro(a)
999. Rehusa contestar

Nota para el entrevistador: *Se necesita un promedio de tiempo de los días en los cuales Usted camina*.
Si la persona entrevistada no puede contestar porque la cantidad de tiempo varía mucho día a día, pregunte: *¿Cuál es la cantidad total de tiempo que Usted pasó caminando en los **últimos 7 días**?"*

__ __ __ Horas por semana [WWHRS; Rango: 0-112]
__ __ __ __ Minutos por semana [WWMIN; Rango: 0-6720, 9998, 9999]
9998. No sabe /no está seguro(a)
9999. Rehúsa contestar

Lea al sujeto: *Ahora piense acerca del tiempo que Usted pasó sentado(a) en la semana durante los últimos 7 días. Incluya el tiempo en el trabajo, en la casa, estudiando y durante el tiempo de descanso. Esto puede incluir tiempo que pasó sentado(a) en un escritorio, visitando amistades, leyendo, sentado(a) o acostado(a) viendo televisión.*

180- ¿Durante los últimos 7 días, ¿Cuánto tiempo en total usted usualmente pasó sentado durante un día en la semana?

__ __ Horas por semana [SDHRS; 0-16]
__ __ __ Minutos por semana [SDMIN; Rango: 0-960, 998, 999]
998. No sabe /no está seguro(a)
999. Rehúsa contestar

Clarificación por parte del entrevistador: *Incluya el tiempo que pasó acostado (despierto) así como sentado*
Nota para el entrevistador: Se necesita un promedio de tiempo al día. Si la persona entrevistada no puede contestar porque la cantidad de tiempo varia día a día, pregunte: "*Cuál fue la cantidad total de tiempo que Usted pasó sentado(a) el **Miércoles** pasado?*"
__ __ Horas el miércoles [SWHRS; Rango 0-16]
__ __ __ Minutos el miércoles [SWMIN; Rango: 0-960, 998, 999]
998. No sabe /no está seguro(a)
999. Rehúsa contestar

di@bet.es
Estudio Epidemiológico de la Diabetes tipo 2 en España

ciberdem
Centro de Investigación Biomédica En Red de Diabetes y Enfermedades Metabólicas Asociadas

FED

SOCIEDAD ESPAÑOLA DE DIABETES

Pegar etiqueta identificativa

DATOS IDENTIFICATIVOS

CÓDIGO: .... EQUIPO:..

NOMBRE .... APELLIDOS …………………………………………………………..………

EXTRACCIÓN Y SOBRECARGA ORAL DE GLUCOSA

CRITERIOS DE EXCLUSIÓN PARA EXTRACCIÓN:

181-¿Ha tenido Ud. fiebre, diarrea o vómitos, en la última semana?

1☐Si, posponer 2☐No 9☐ns/nc

182- ¿A qué hora ha comido o bebido por última vez? (salvo agua)**:**

....

183- Horas de ayuno: ....horas (si menos de 8h postponer)

CRITERIOS DE EXCLUSIÓN PARA SOG:

184- ¿Es Ud. diabético? 1☐Si 2☐No 9☐ns/nc

185- ¿Toma Insulina? 1☐Si (no realizar SOG) 2☐No 9☐ns/nc

186- ¿Toma Ud. alguna pastilla para controlar el azúcar?

1☐Si (no realizar SOG) 2☐No 9☐ns/nc

187- ¿A qué hora tomó la última?: ....

188- Fecha de la extracción (dd/mm/aa): ....

189- Problemas y observaciones: ....

....

....

....

190- Glucemia capilar basal: .... mg/dl **192- Hora**: ....

(Si es mayor de 180, repetir y si se confirma, no realizar la SOG)

191- Glucemia postSOG (2h): .... mg/dl .**193Hora**: ....

194 ¿Se obtiene muestra venosa basal? 1☐Si 2☐No

195 ¿Se obtiene muestra venosa post SOG? 1☐Si 2☐No
