# Supplementary material for: Low Physical Activity and Its Association with Diabetes and Other Cardiovascular Risk Factors: A Nationwide, Population-Based Study
Source: PLoS One. 2016 Aug 17;11(8):e0160959. doi: 10.1371/journal.pone.0160959 (PMC4988632; doi:10.1371/journal.pone.0160959)
Supplement: S2 File — (PDF) [file pone.0160959.s002.pdf]

**di@bet.es**
Estudio Epidemiológico de la Diabetes tipo 2 en España

ciberdem
Centro de Investigación Biomédica En Red de Diabetes y Enfermedades Metabólicas Asociadas

Paste identification tag

## IDENTIFICATION DATA

CODE: ....................................DATE: ............................. GROUP: ..................................

NAME: ............................................................................... LAST NAME(S): …………………………………………………………………………………………

ADDRESS: ...........................................................................................ZIP CODE:…..........TOWN/ CITY: ...................................STATE: .................................

PHONE: ...........................................................DATE OF BIRTH: .............................. CURRENT AGE: .................. ☐ MALE ☐ FEMALE

COUNTRY OF BIRTH: .......................................................ETHNICITY: ...................................................INTERVIEWER: ……………………………………

## EXCLUSION CRITERIA

| | **Indicate whether any of the criteria are met:** |
|---|---|
| 1- Do you currently live in a hospital, nursing home or assisted living center?<br>1☐ yes 2 ☐ NO 9☐ ns, nc* | ☐ EXCLUDED |
| 2- Are you pregnant?<br>1☐ YES 2☐ NO 9 ☐ ns, nc | ☐ EXCLUDED |
| 3- Have you had a child in the last 6 months?<br>1 ☐ YES 2☐ NO 9☐ ns, nc | ☐ EXCLUDED |
| 4- Have you undergone any major surgery in the last month?<br>1 ☐ YES 2☐ NO 9☐ ns, nc | ☐ EXCLUDED |
| 5- Have you suffered any serious illnesses not related to diabetes in the last 6 months?<br>1☐ YES 2☐ NO 9☐ ns, nc | ☐ EXCLUDED |

***ns, nc: does not know; does not answer**

**If excluded, go to page 4: Known diabetes, hypertension and cardiovascular disease**

## SOCIAL STATUS

| 6- Civil status | 7- What is your highest level of studies attained? | 8- Do you have a job? |
|---|---|---|
| 1☐ Single<br>2☐ Married OR domestic partnership<br>3☐ Widowed<br>4☐ Divorced<br>9☐ ns / nc | 1☐ Cannot read/write<br>2 ☐ No formal schooling<br>3 ☐ Primary education (up to 12 years)<br>4☐ Secondary education (16 years)<br>5☐ Bachelor, technical training (18 years)<br>6☐ University first cycle studies (associate's degree program)<br>7☐ University second cycle studies (undergraduate degree program, engineering, medicine)<br>9☐ ns / nc | 1☐ YES 2☐ NO 9☐ ns / nc<br>**9- If you do not work, what is the reason?**<br>1☐ Retired<br>2☐ Unemployed<br>3 ☐ Sick leave<br>4☐ Student<br>5☐ Housewife<br>9☐ ns / nc |

**10- What is your current occupation or what was your main occupation for the majority of your life? Explain ……………………………………………**

1☐ Executives of public or private companies with 10 or more employees.
Professions associated with university second cycle or postgraduate studies.
2☐ Directors of companies with fewer than 10 employees.
Professions associated with university first cycle studies.
Senior Technicians. Artists and athletes.
3☐ Administrative employees, management and support personnel.
Workers in personal services and security.
Free-lance workers.
Supervisors of manual workers.

4 ☐ Skilled manual workers.
5 ☐ Semi-skilled manual workers.
6 ☐ Unskilled workers.
7 ☐ Other (clergy, military, ...).

## SMOKING HABITS

**11- Do you smoke?**

1☐ Yes 2☐ No 9☐ns/nc

**12- How many cigarettes do you smoke? ..................../day**

**13- At what age did you start smoking? ....................**

**14- Have you changed your smoking habits in the last 6 months?**

1☐ Yes, I smoke more 3☐ Yes, I smoke less

2☐ NO 9☐ns / nc

**IF NOT CURRENTLY A SMOKER**

**15- Have you ever smoked more than 1 cig./day at some point during your life?**

1☐Yes 2☐No 9 ☐ns/nc

**16- How much did you use to smoke? ................**

**17- At what age did you quit smoking? ..........**

**18- At what age did you start smoking? ..........**

## PHYSICAL ACTIVITY

**19- How many hours do you usually sleep? ................ hours / day**

**20- Which of these options best describes your main activity during the day, while at work, school, or home?**

1 ☐ Sitting most of the day

2 ☐ Standing most of the day without significant movement and/or effort

3 ☐ Walking, carrying some weight, or making frequent trips that do not require great physical effort

4 ☐ Performing tasks that require a great amount of physical effort

**21- In your free time, do you usually perform heavy physical exercise at least once a week?**

1☐Yes 2☐No 9☐ns /nc

**If the answer is yes:**

**22-** How many days a week? ............days

**23-** On the days that you exercise, for how long do you exercise? .......... hours

**24-** What kind of exercise do you do? ............................................

## HISTORY OF DIABETES

**25 - Have you ever been told by a doctor that you had diabetes? (sugar in blood)**

1☐Yes 2☐No 9☐ns/nc

**26- If yes, how old were you at the time of diagnosis? .................years old**

**27- Do any of your relatives (parents, siblings, children, or grandparents) have diabetes?**

1☐Yes 2☐No 9☐ns/nc

**Specify how many:**

28- Grandparents: .........

29- Parents: .........

30- Uncles and aunts: .........

31- Brothers and sisters: .........

32- Children**:** .........

**33- Do you take any pills for diabetes?**

1☐Yes 2☐No 9☐ns/nc

**34- If yes, for how long have you been taking them? .......years**

**35- Are you injecting insulin?**

1☐Yes 2☐No 9☐ns/nc

**36- If yes, for how long have you been injecting insulin? .......years**

**37- Have you ever been on a diet for your diabetes, as guided by your doctor?**

1☐Yes 2☐No 9☐ns/nc

**38- If yes, for how long have you been following the diet? ........years***(If not following any diet for diabetes, mark 77.)*

## HISTORY OF ARTERIAL HYPERTENSION

**39- Have you ever been told by a doctor that you had high blood pressure?** 1☐Yes 2☐ No 9☐ ns/nc

**40- If yes, how old were you when he/she told you that you had hypertension? ....................years old**

**41- Do you take any medication for hypertension?** 1☐Yes 2☐ No 9☐ ns/nc

**42- If yes, for how long have you been taking it? .............years**

**43- Have you taken it in the last 24 hours?** 1☐ Yes 2☐ No 9☐ ns/nc

## CARDIOVASCULAR AND OTHER DISEASES

**44- Have you ever been told by a doctor that you had high cholesterol?**

1☐Yes 2☐No 9☐ns/nc

**45- Have you been told by a doctor that you have suffered a heart attack, angina or myocardial infarction?**

1☐Yes 2☐No 9☐ns/nc

**46- Have you been told by a doctor that you have suffered a stroke, cerebral hemorrhage or cerebral palsy?**

1☐Yes 2☐No 9☐ns/nc

**47- Have you ever been told by a doctor that you have peripheral vascular disease (foot ulcers)?**

1☐Yes 2☐No 9☐ns/nc

**48- Have you been told by a doctor that you suffer from a chronic disease (bronchitis, asthma, rheumatism, tumors etc)?**

1☐Yes 2☐No 9☐ns/nc **49-If you answered yes to any of the above questions, explain:** ......................................................

**Drugs that you regularly take, including vitamins, analgesics, tranquilizers, ... etc. (Not including herbal products)**

| | |
|---|---|
| 50............................................................................................................. | 55............................................................................................................. |
| 51............................................................................................................. | 56............................................................................................................. |
| 52............................................................................................................. | 57............................................................................................................. |
| 53............................................................................................................. | 58............................................................................................................. |
| 54............................................................................................................. | 59............................................................................................................. |

## GYNECOLOGICAL HISTORY

**60- How many childbirths have you had? .......... Weights of children at birth**: .............kg, .............kg, .............kg, .............kg, .............kg, ............kg,

**61- Were any of your pregnancies interrupted? If so, how many?** ...................................

**62- Had your doctor ever told you that you had gestational diabetes?**

1☐Yes 2☐No 9☐ns/nc **63- If yes, in how many pregnancies?** ......................................................

**64- How old were you when you had your first menstrual period? .......... years old**

**65- Do you use hormonal contraceptives? (Oral or patch)**

1☐ Yes 2☐No 9☐ns/nc If yes, which one do you use? ......................................................

**66- If yes, for how long have you been taking them (regardless of how many times the composition may have been changed)? ............. years**

**67- What was the date of your last menstrual period? ................**

**68- If you have had amenorrhea (lack of menstrual period) for 6 months or longer, what was the cause?**

1☐Natural 2☐Surgical ☐ns/nc

**69- Are you on treatment for menopause?**

1☐ Yes 2☐ No 9☐ns/nc **70- If yes, specify:** ..................................................................................................................................

**71- If yes, for how long have you been on this treatment (regardless of how many times the composition may have been changed)? ............. years**

## WEIGHT CHANGES

**72- Do you remember how much you weighed at age 18? .......................**

**73- Do you weigh more now than when you were 18?**
1☐Yes 2☐ No, the same 3☐No, less weight 9☐ns/nc

**74- Have you lost weight in the past year?**
1☐Yes 2☐No 9☐ns/nc

**75- If yes, how many kg you have you lost? .............. kg**

**76- Was this loss involuntary?**
1☐Yes 2☐No 9☐ns/nc

**77- Have you gained weight in the past year?**
1☐Yes 2☐No 9☐ns/nc

**78- If yes, how many kg did you gain? ............... kg**

**79- Would you like to ...?**
1☐ Gain Weight 2☐ Lose Weight 3☐ Maintain the same weight

**80- In your opinion you are:**
1☐ Thin 2☐ Normal
3☐ Overweight 4☐ Obese 9☐ns/nc

## ANTHROPOMETRICS AND BLOOD PRESSURE

**81- Weight**: .................. kg

**82- Height**: ..................cm

**83- Waist** : ................cm

**84- Hip:** ..................cm

**85- Systolic blood pressure 1**: ..................mmHg

**86- Diastolic blood pressure 1**: ................mmHg

**87- Systolic blood pressure 2**: ..................mmHg

**88- Diastolic blood pressure 2**: ................mmHg

**If the values show a difference of >5 mmHg, a 3rd measurement will be taken:**

**89- Systolic blood pressure 3**: ..................mmHg

**90- Diastolic blood pressure 3**: ................mmHg

**91- If any measurement cannot be taken, indicate which and why:**

..........................................................................................................

## OBSERVATIONS

## DIETARY HABITS

**92- Have you changed the amount you use to eat in the last 6 months?**
1☐ Yes, I eat more 3☐ Yes, I eat less
2☐ No, I eat the same 9☐ ns/nc

**93- Have you changed the type of food in your diet over the past 6 months?**
1☐Yes 2☐No 9☐ns/nc

**94- If yes, specify the change: ………………................……………….**

**95- If yes, was the change recommended by a doctor or dietitian?**
1☐ Yes 2☐No 9☐ns/nc

**96- If you are on a diet, specify what kind of diet you are following:** ....................................................
..........................................................................................................................

**97- How many times do you eat out per week?**
1 ☐ daily
2 ☐ from 6 to 4 per week
3 ☐ from 3 to 2 per week
4 ☐ once per week
5 ☐ 3 to 2 times a month
6 ☐ once a month
7 ☐ seldom

**98- Do you take any kind of vitamin supplement?**
1 ☐ Yes 2 ☐ No 9 ☐ ns / nc, **If yes, specify:** ....................

**99- Do you use saccharin or any kind of non-caloric sweetener?**
1☐Yes 2☐No 3☐ns/nc 100- Specify .............................

**100.B- Do you usually use fructose?**
1☐Yes 2☐No 3☐ns/nc

**100.C- What type of salt do you use?**
1☐ Iodized 2☐Sea 3☐Others 4☐ns/nc

**101- How many meals you usually have in a typical day?**
1☐ less than 3
2☐ 3
3☐ 4 or 5
4☐ more than 5

**102- Do you snack between meals?**
1☐ Yes 2☐ No 3☐ Sometimes 9☐ns/nc

**103- Do you eat while watching TV?**
1☐ Yes 2☐ No 3☐ Sometimes 9☐ns/nc

**104- What do you eat while watching TV?**
1☐ Meals
2☐ Snacks
3☐ Both

In each row, mark the box that best describes how often each type of food is consumed:

| | | PER DAY | | | | | PER WEEK | | | PER MONTH | | SELDOM OR NEVER |
|---|---|---|---|---|---|---|---|---|---|---|---|---|
| | | 5 times or more | 4 times | 3 times | 2 times | 1 time | 4-6 times | 2-3 times | 1 time | 2-3 times | 1 time | |
| **105-** | **Breakfast cereals** | | | | | | | | | | | |
| **106-** | **Whole grain breakfast cereals** | | | | | | | | | | | |
| **107-** | **White bread** | | | | | | | | | | | |
| **108-** | **Whole wheat bread** | | | | | | | | | | | |
| **109-** | **White rice, pasta or couscous** | | | | | | | | | | | |
| **110-** | **Brown rice or whole wheat pasta or couscous** | | | | | | | | | | | |
| **111-** | **Sugar, honey, candies, jams** | | | | | | | | | | | |
| **112-** | **Chocolate** | | | | | | | | | | | |
| **113-** | **Sweets, cakes, muffins, cookies, donuts** | | | | | | | | | | | |

| | PER DAY | | | | | PER WEEK | | | PER MONTH | | SELDOM OR NEVER |
|---|---|---|---|---|---|---|---|---|---|---|---|
| | 5 times or more | 4 times | 3 times | 2 times | 1 time | 4 - 6 times | 2 - 3 times | 1 time | 2 - 3 times | 1 time | |
| **114- Eggs** | | | | | | | | | | | |
| **115- Milk, yogurt, cheese** | | | | | | | | | | | |
| **116- Milk shakes, flan, custard, ice cream** | | | | | | | | | | | |
| **117- Cream, butter** | | | | | | | | | | | |
| **118- Margarine** | | | | | | | | | | | |
| **119- Mayonnaise** | | | | | | | | | | | |
| **120- Homemade fried food** | | | | | | | | | | | |
| **121- Precooked foods (fried / frozen)** | | | | | | | | | | | |
| **122- Other precooked dishes (including pizza)** | | | | | | | | | | | |

In each row, mark the box that best describes how often each type of food is consumed:

| | PER DAY | | | | | PER WEEK | | | PER MONTH | | SELDOM OR NEVER |
|---|---|---|---|---|---|---|---|---|---|---|---|
| | **5 times or more** | **4** times | **3** times | **2** times | **1** time | **4 - 6** times | **2 - 3** times | **1** time | **2 - 3** times | **1** time | |
| **123- Salads (raw vegetables)** | | | | | | | | | | | |
| **124- Vegetable side✳** | | | | | | | | | | | |
| **125- Vegetables in stew✳✳** | | | | | | | | | | | |
| **126- Legumes** | | | | | | | | | | | |
| **127- Potato: stewed, boiled or fried at home (NO crisps).** | | | | | | | | | | | |
| **128- Fruits / natural juices** | | | | | | | | | | | |
| **129- Nuts and similar** | | | | | | | | | | | |
| **130- Appetizers: cheese puffs, corn snack food, popcorn etc.** | | | | | | | | | | | |
| **131- Fish / fresh seafood** | | | | | | | | | | | |
| **132- Canned fish / seafood** | | | | | | | | | | | |
| **133- Meat, pork, beef, chicken...** | | | | | | | | | | | |
| **134- Cured, dry sausages, ham** | | | | | | | | | | | |

**✳Side = vegetable(s) that is/are consumed as such, as a main dish or accompanying a 2nd course, but not used to prepare stews, sautees, etc.**

**✳✳ Vegetables consumed with lentils, stews, stir-fries, etc., and of which only a small amount is used in each dish.**

**What kind of oil do you usually use? (Multiple answers may be marked for each item.)**

| | **1- Olive oil** | **2- Linoleic sunflower** | **3- High oleic sunflower** | **4- Others** |
|---|---|---|---|---|
| 135- To season (salads, etc) | | | | |
| 136- To stew | | | | |
| 137- To fry | | | | |
| 138- To prepare mayonnaise | | | | |

## ALCOHOLIC BEVERAGES

| | **PER DAY** | | | | | **PER WEEK** | | | **PER MONTH** | | **SELDOM OR NEVER** |
|---|---|---|---|---|---|---|---|---|---|---|---|
| | **5 times or more** | **4** times | **3** times | **2** times | **1** time | **4 - 6** times | **2 - 3** times | **1** time | **2 - 3** times | **1** time | |
| 139- Red Wine | | | | | | | | | | | |
| 140- White wine, sparkling wine | | | | | | | | | | | |
| 141- Rose wine | | | | | | | | | | | |
| 142- Dessert wine | | | | | | | | | | | |
| 143- Beer | | | | | | | | | | | |
| 143b- Cider | | | | | | | | | | | |
| 144- Distilled spirits | | | | | | | | | | | |

**145- When you drink alcohol?**
1☐ With meals
3☐ Both
2☐ Without food/meals
9☐ ns/nc

**146- Frequency**
1☐ Daily or almost daily
3☐ occasionally
2☐ On weekends
4☐ never
9☐ns/nc

## NON-ALCOHOLIC BEVERAGES

| | PER DAY | | | | | PER WEEK | | | PER MONTH | | SELDOM OR NEVER |
|---|---|---|---|---|---|---|---|---|---|---|---|
| | **5 times or more** | **4** times | **3** times | **2** times | **1** time | **4 - 6** times | **2 - 3** times | **1** time | **2 - 3** times | **1** time | |
| 147- Beer without alcohol | | | | | | | | | | | |
| 148- Beverages with added sugar | | | | | | | | | | | |
| 149- Beverages with no added sugar (light or diet) | | | | | | | | | | | |
| 150- Commercial juices | | | | | | | | | | | |
| 151- Caffeinated soft drinks | | | | | | | | | | | |
| 152- Espresso | | | | | | | | | | | |
| 153- Brewed coffee | | | | | | | | | | | |
| 154- Instant coffee | | | | | | | | | | | |
| 155- Decaffeinated brewed coffee | | | | | | | | | | | |
| 156- Decaffeinated instant coffee | | | | | | | | | | | |
| 157- Tea (specify) | | | | | | | | | | | |
| 158- Other infusions (specify) ....................................................... ....................................................... | | | | | | | | | | | |

**159- Is the person who normally prepares the consumed foods/beverages present at the time of the interview?** 1☐Yes 2☐No 9☐ns/nc

**INTERVIEWER'S NOTES:**
**160- Level of interest:** 1☐ High 2 ☐ Average 3 ☐ Low

**161- Accuracy in answering:** 1 ☐ Good 2 ☐ Fair 3 ☐ Poor

**QUALITY OF LIFE QUESTIONNAIRE** (Questionnaire SF-***12) (TRANSLATED FROM THE SPANISH VALIDATED VERSION)***

*The following questions refer to your perception of health and well-being. Your answers will enable us to know how capable you see yourself of performing routine activities. If you are not sure how to answer a question, choose the most appropriate answer from the options provided.*

**162- In general, you would say that your health is:**

1☐ Excellent 2☐ Very good 3☐ Good 4☐ Fair 5☐ Poor

**Read to the subject:** *The following questions are about activities that you might do during a typical day. Does your health currently limit your capacity to perform these activities? If so, how much?*

**163- Moderate activities, such as moving a table, pushing a vacuum cleaner or walking 1 hour**

1-☐ YES, very limited 2- ☐ YES, somewhat limited 3-☐ NO, not limited at all

**164- Climbing several flights of stairs.**

1-☐ YES, very limited 2-☐ YES, somewhat limited 3-☐ NO, not limited at all

**Read to the subject:** *During the past 4 weeks, have you had any of the following problems performing work or other regular daily activities, as a result of your physical health?*

**165- Accomplished less than you would like.** 1☐Yes 2☐No

**166- Limited in the kind of work or other activities performed.** 1☐Yes 2☐No

**Read to the subject:** *During the past 4 weeks, have you had any of the following problems performing work or other regular daily activities, as a result of* ***emotional problems*** *(such as feeling depressed or anxious)?*

**167- Accomplished less than you would like.** 1☐Yes 2☐No

**168- Performed work or activities with less care than usual.** 1☐Yes 2☐No

**169- During the past 4 weeks, did you have any pain?** ☐ Yes ☐ No

**169.B During the past 4 weeks, how much did your pain interfere with your normal work (including work other than housework)?**

1☐ Not at all 2☐ A little bit 3☐ Some 4☐ Quite a bit 5☐ A lot

**Read to the subject:** *These questions are about how you have been feeling during the past 4 weeks.*

*For each question, please give the one answer that best reflects the way you have been feeling.*

*How much of the time* ***during the past 4 weeks…***

**170- Have you felt calm and peaceful?**

1☐ All the time 2☐ Most of the time 3☐ A good bit of the time 4☐ Some of the time 5☐ A little of the time 6☐ None of the time

**171- Did you have a lot of energy?**

1☐ All the time 2☐ Most of the time 3☐ A good bit of the time 4☐ Some of the time 5☐ A little of the time 6☐ None of the time

**172- Have you felt discouraged and/or upset?**

1☐ All the time 2☐ Most of the time 3☐ A good bit of the time 4☐ Some of the time 5☐ A little of the time 6☐ None of the time

**173- During the past 4 weeks, how much of your time has your physical health or emotional problems interfered with social activities (like visiting friends, relatives, etc.)?**

1☐ All the time 2☐ Most of the time 3☐ A good bit of the time 4☐ Some of the time 5☐ A little of the time 6☐ None of the time

**PHYSICAL ACTIVITY QUESTIONNAIRE** (IPAQ)

**Read to the subject**: *Now I'll ask you about the time you spent being physically active during the last 7 days. Please answer each question even if you consider yourself to be an inactive person. Think about the activities you do at work, around the house and in the yard, to get from place to place, and in your spare time, for recreation, exercise or sport*.

**Read the subject:** *Now, think about all the vigorous activities that you did during the last 7 days. Vigorous physical activities refer to activities that take hard physical effort and make you breathe much harder than normal. Think only about those physical activities that you did for at least 10 minutes at a time.*

**174- During the last 7 days, on how many days did you do vigorous physical activities?**

_____ Days per week [VDAY; Range: 0 - 7, 8 ,9]
8. Does not know / is not sure
9. Refuses to answer

**Interviewer clarification:** *Think only about those physical activities that you do for at least 10 minutes.*
**Interviewer Note:** *If respondent answers zero, refuses to answer or does not know, skip to question 176.*

**175- How much time did you usually spend doing vigorous physical activities on one of those days?**

__ __ Hours per day [VDHRS; Range: 0-16]
__ __ __ Minutes per day [VDMIN; Range: 0-960, 998, 999]
998. Does not know / is not sure
999. Refuses to answer

**Interviewer clarification:** *Think only about those physical activities that you do for at least 10 minutes.*
**Interviewer note:** *We are looking for an average amount of time per day. If the respondent cannot answer because the amount of time varies from day to day, ask: "How long did you spend total performing vigorous physical activities during the last 7 days?"*

__ __ Hours per week [VWHRS; Range: 0-112]
__ __ __ __ Minutes per week [VWMIN; Range: 0-6720, 9998, 9999]
9998. Does not know / is not sure
9999. Refuses to answer

**Read the subject:** *Now think about all the moderate activities that you did in the last 7 days. Moderate activities refer to activities that take moderate physical effort and make you breathe somewhat harder than normal. Think only about those physical activities that you did for at least 10 minutes at a time.*

**176- During the last 7 days, on how many days did you do moderate physical activities?**

____ Days per week [MDAY; Range: 0-7, 8, 9]
8. Does not know / is not sure
9. Refuses to answer

**Interviewer clarification:** *Think only about those physical activities that you do for at least 10 minutes.*
**Interviewer Note:** *If respondent answers zero, refuses or does not know, skip to question 178.*

**177- How much time did you usually spend doing moderate physical activities on one of those days?**

__ __ Hours per day [MDHRS; Range: 0-16]
__ __ __ Minutes per day [MDMIN; Range: 0-960, 998, 999]
998. Does not know / is not sure
999. Refuses to answer

**Interviewer clarification:** *Think only about those physical activities that you do for at least 10 minutes.*
**Interviewer note:** *An average amount of time per day on one of the days on which you did moderate physical activity is needed. If the respondent cannot answer because the amount of time varies from day to day, or includes time spent in different jobs, ask: "How much time did you devote to doing moderate physical activity during the last 7 days"?*

__ __ __ Hours per week [MWHRS; Range: 0-112]
__ __ __ __ Minutes per week [MWMIN; Range: 0-6720, 9998, 9999]
9998. Does not know / is not sure
9999. Refuses to answer

**Read the subject:** *Now think about the time you spent walking in the last 7 days. This includes at work and at home, walking to travel from place to place, and any other walking that you have done solely for recreation, sport, exercise, or leisure.*

**178- During the last 7 days, on how many days did you walk for at least 10 minutes at a time?**

____ Days per week [WDAY; Range: 0-7, 8, 9]
8. Does not know / is not sure
9. Refuses to answer

**Interviewer clarification:** *Think only about the walking that you did for at least 10 minutes at a time.*
**Interviewer Note:** *If respondent answers zero, refuses or does not know, skip to question 180.*

**179- How much time did you typically spend walking on one of those days?**

__ __ Hours per day [WDHRS; Range: 0-16]
__ __ __ Minutes per day [WDMIN; Range: 0-960, 998, 999]
998. Does not know / is not sure
999. Refuses to answer

**Note to interviewer:** *an average amount of time per day spent walking is needed.*
**If the respondent cannot answer because the amount of time varies greatly from day to day, ask: What is the total amount of time you spent walking in the last 7 days"?**

__ __ __ Hours per week [WWHRS; Range: 0-112]
__ __ __ __ Minutes per week [WWMIN; Range: 0-6720, 9998, 9999]
9998. Does not know / is not sure
9999. Refuses to answer

**Read the subject:** *Now think about the time you spent sitting on weekdays during the last 7 days. Include time spent at work, at home, while studying and during leisure time. This may include time spent sitting at a desk, visiting friends, reading, or sitting and/or lying down (awake) to watch television.*

**180- During the last 7 days, how much time did you spend on average sitting on a week day?**

__ __ Hours per week [SDHRS; 0-16]
__ __ __ Minutes per week [SDMIN; Range: 0-960, 998, 999]
998. Does not know / is not sure
999. Refuses to answer

**Interviewer clarification:** *Include time spent lying down (awake) as well as sitting.*
**Interviewer note: An average amount of time per day is needed. If the respondent cannot answer because the amount of time varies day to day, ask: "What was the total amount of time you spent sitting (a) last Wednesday?"**

__ __ Hours last Wednesday [SWHRS; Range 0-16]
__ __ __ Minutes last Wednesday [SWMIN; Range: 0-960, 998, 999]
998. Does not know / is not sure
999. Refuses to answer

**di@bet.es**
Estudio Epidemiológico de la Diabetes tipo 2 en España

Paste identification tag

## IDENTIFICATION DATA

CODE: ............................................. GROUP: ...............................................

FIRST NAME ........................................................ LAST NAME(S): ……………………………………………………………..………

**BLOOD SAMPLE AND ORAL GLUCOSE TEST TOLERANCE (OGTT)**

**EXCLUSION CRITERIA FOR BLOOD SAMPLE:**

**181- Have you experienced fever, diarrhea or vomiting in the last week?**

1☐ Yes (postpone) 2☐No 9☐ns/nc

**182- When was the last time you had something to eat or drink (other than water)?:** ....................................................

**183- How many hours spent fasting:** …… hours (if less than 8h, postpone)

**Exclusion criteria for OGTT:**

**184- Do you have diabetes?** 1☐Yes 2☐No 9☐ns/nc

**185- Do you take insulin?** 1☐ Yes (not perform OGTT) 2☐No 9☐ns/nc

**186- Do you take pills for sugar/glycemic control?**

1☐ Yes (not perform OGTT) 2☐No 9☐ns/nc

**187- When was the last time you took one?:**..............

**188- Blood sample date (dd / mm / yy):**..........................

**189- Problems and observations:**...............................................................

.....................................................................................................................

.....................................................................................................................

.....................................................................................................................

**190- Capillary basal glycemia**: ............. mg/dl **192- Hour**: .........................

(If higher than 180, repeat and, if confirmed, do not perform OGTT)

**191- Glycemia post-OGTT (2h):** ............. mg/dl **193- Hour**: .........................

**194- Basal venous sample obtained?** 1☐Yes 2☐No

**195- Post-OGTT venous sample obtained?** 1☐Yes 2☐No
